# Early Education on Intimate Partner Violence in Undergraduate Medical Training: A Narrative Review on Timing, Methods, and Clinical Preparedness

**DOI:** 10.7759/cureus.112452

**Published:** 2026-07-11

**Authors:** Christina Bedley, Laura Menendez, Madeline Culbreth, Lauren Fine

**Affiliations:** 1 College of Allopathic Medicine, Nova Southeastern University Dr. Kiran C. Patel College of Allopathic Medicine, Lauderdale, USA; 2 College of Allopathic Medicine, Nova Southeastern University Dr. Kiran C. Patel College of Allopathic Medicine, Fort Lauderdale, USA

**Keywords:** intimate partner violence (ipv), ipv screening, simulation in medical education, trauma informed care, undergraduate medical training

## Abstract

Intimate partner violence (IPV) encompasses physical, emotional, and sexual abuse within intimate relationships and is commonly encountered in clinical practice, affecting individuals across all backgrounds. Globally, healthcare settings represent a critical point of contact for individuals who have experienced intimate partner violence. IPV is associated with adverse health outcomes including depression, post-traumatic stress disorder, substance use disorders, traumatic brain injury, and adverse pregnancy outcomes. Despite high prevalence and recommendations to routinely screen women of reproductive age, IPV screening rates remain inconsistent, highlighting the need to examine how IPV education is incorporated into medical training. This narrative review evaluates undergraduate medical education on IPV, focusing on timing, instructional methods, and impact of interventions on student preparedness. A search was conducted in PubMed and Embase for studies published between 2000 and 2025, from which eligible articles were selected for full-text review. Existing curricula use multimodal approaches, including didactic sessions, small-group discussions, and simulation-based training, but are often isolated, optional, and rarely reinforced longitudinally. While these approaches improve short-term knowledge and confidence, gaps remain in sustained preparedness and consistent screening practices. Current approaches to IPV education may overestimate preparedness by emphasizing self-reported, short-term confidence, while failing to evaluate whether skills are consistently applied in real-world clinical settings. Repeated exposure to trauma-informed IPV education during preclinical years may help normalize screening and strengthen recognition skills. Future strategies should prioritize longitudinal, community-based curricula that reinforce IPV screening and response across multiple stages of medical training to better prepare physicians for real-world patient care.

## Introduction and background

Intimate partner violence (IPV) is a common and serious issue physicians encounter during their clinical practice. IPV includes any sexual, physical, or emotional abuse that occurs across genders, sexual orientations, and socioeconomic backgrounds. According to the World Health Organization, an estimated one in three women worldwide have experienced some form of IPV in their lifetime [1].

Importantly, IPV is prevalent across the reproductive lifespan and is especially relevant in pregnancy and antenatal care. Pregnancy represents a particularly high-risk period, with evidence suggesting that approximately 25% of women worldwide experience some form of IPV during pregnancy [2]. IPV also frequently intersects with acute care: one study reported that 44% of women later killed by an intimate partner had visited an emergency department in the year prior to their death, many for IPV-related injuries. However, IPV was rarely identified or directly addressed during those encounters [1]. Disparities in IPV prevalence are further pronounced across socioeconomic and geographic contexts, with studies from low-resource settings demonstrating even higher rates of IPV exposure. This highlights the role of structural and social determinants in shaping risk of experiencing IPV [3]. These patterns indicate that IPV is prevalent worldwide and concentrated within clinical contexts that physicians routinely encounter, further reinforcing its relevance to everyday medical practice.

Beyond its high prevalence, IPV is associated with substantial acute and chronic health consequences that frequently bring survivors into contact with the healthcare system. Mental health outcomes are among the most well-documented, including elevated rates of depression, anxiety, and substance use disorders [3]. Physical and neurologic consequences are also common, with IPV-related traumatic brain injury (TBI) reported in a substantial proportion of survivors. IPV-related TBI has been associated with chronic pain, headaches, dizziness, and persistent cognitive symptoms [4]. In reproductive contexts, IPV during pregnancy has been associated with adverse maternal and obstetric outcomes, including miscarriage, preterm birth, and increased maternal mortality risk [2]. Collectively, these mental, physical, and reproductive health effects contribute to increased healthcare utilization, including frequent emergency department visits and healthcare encounters [1]. As a result, healthcare professionals may interact with IPV survivors repeatedly across healthcare settings, creating opportunities for IPV identification and intervention that may otherwise be missed without adequate IPV screening and recognition.

Despite clear clinical guidelines and frequent patient contact, IPV remains substantially under-identified across healthcare settings. The U.S. Preventive Services Task Force (USPSTF) recommendations support routine IPV screening for women of reproductive age, yet screening rates in clinical practice remain inconsistent [5,6]. This gap may partially reflect that IPV often does not present as an isolated traumatic event, but rather through repeated visits, chronic pain complaints, mental health concerns, or substance use [7]. This pattern is particularly relevant in emergency and primary care settings, where survivors may present multiple times without disclosing abuse. Because leaving an abusive relationship can be a high-risk period and many victims never report IPV to police, healthcare settings, especially the emergency department, may represent a critical point of entry for identification and support [1].

Real-world data demonstrates that opportunities for IPV identification are often missed in clinical practice. Although the USPSTF recommends routine IPV screening for women of reproductive age, IPV remains under-recognized in healthcare settings [5]. In a retrospective review of annual primary care visits, IPV screening occurred in only 8.5% of encounters, substantially lower than screening rates for depression and anxiety despite comparable USPSTF Grade B recommendations for routine screening [6]. Additionally, 154 patients presented with symptoms potentially consistent with IPV, yet only nine were appropriately screened, highlighting substantial missed opportunities for identification [5]. Collectively, these findings demonstrate a persistent gap between guideline recommendations and real-world clinical practice.

Beyond individual provider barriers, system-level factors also influence whether IPV screening is implemented and sustained. Studies of IPV screening programs within large healthcare systems, such as the Veterans Health Administration, demonstrate that sustained screening requires institutional support, clear protocols, leadership engagement, and ongoing training [8]. In the absence of these supports, IPV screening is often inconsistently applied, deprioritized, or abandoned over time. Together, these findings underscore that gaps in IPV identification reflect not a lack of clinical opportunity, but insufficient preparation, training, and structural support, highlighting the need to examine how IPV education is delivered during medical training. Importantly, existing literature largely evaluates IPV education using self-reported confidence and knowledge, with limited assessment of whether these skills translate into clinical application or sustained screening practices. This gap highlights a critical disconnect between educational outcomes and real-world patient care [9].

This review highlights the critical importance of training healthcare providers to identify and appropriately intervene in cases of IPV. Such training can be introduced during medical student years through trauma-informed care education and integration of IPV-related clinical scenarios into the curriculum. Trauma-informed care is a model in which the provider recognizes, assesses, and treats patients who have experienced traumatic events. As part of this framework, the provider must seek to understand how they can help foster a stable relationship with the patient and understand their background. These principles apply to IPV screening, considering the importance of healthcare providers being aware of all possible presentations of a patient who is experiencing IPV. Physician-patient interactions should foster this level of comfort and communication to ensure the best outcomes in screening and treatment [10]. Early exposure to recognizing and counseling patients affected by IPV may better prepare students to effectively respond as physicians in clinical settings.

The purpose of this narrative review is to evaluate existing IPV education in undergraduate medical training, with emphasis on how preparedness is measured and the potential benefits of repeated, longitudinal exposure. This study offers a novel perspective on the timing of IPV educational intervention within the undergraduate medical curriculum, including longitudinal, community-based approaches to IPV educational training.

## Review

Methods

We conducted a narrative review to synthesize existing evidence regarding IPV education in undergraduate medical training. Undergraduate medical training encompasses both the preclinical and clinical components of the medical school curriculum. A literature search was performed using Embase and PubMed in 2025. Search terms included combinations of keywords such as “intimate partner violence”; “medical education”; “trauma-informed care”; “screening”; “undergraduate medical education”; “simulation”. Articles published between 2000 and 2025 were considered for inclusion. 284 articles were identified, and 159 were screened after removing duplicates. Following abstract review, 21 studies underwent full-text evaluation for relevance to undergraduate IPV education, curricular structure, preparedness outcomes, and screening practices. Articles were excluded if they were outside the United States, focused primarily on residents rather than undergraduate medical students, or did not align with the aims of the review.

Although IPV is a global public health issue, this review was limited to US-based studies because undergraduate medical curricula vary substantially across countries, making direct comparisons of educational interventions and outcomes limited. Further, the review focused on US medical schools because the American Medical Association and the Association of American Medical Colleges have established recommendations for IPV education integration, providing a consistent policy framework for evaluating curriculum implementation. After exclusion, six articles were analyzed by accounting for results that demonstrated quantifiable data, including surveys of retention rates, formal simulation assessments, and narrative feedback from students who participated in IPV curriculum as part of their medical school education. Additional epidemiologic, screening, and systems-based literature was incorporated to provide clinical and educational context. These articles are not referenced in the results section.

Results

Overview of Included Literature

Despite recognition of IPV as a major public health issue, existing literature suggests that IPV education within health and professional training programs remains insufficiently integrated and prioritized, resulting in persistent preparedness gaps among medical trainees.

Current IPV Educational Approaches

The current literature demonstrates that undergraduate medical education IPV curricula commonly utilize a multimodal instructional approach, including didactic lectures, small-group discussions, and simulations using standardized patient encounters, with occasional reinforcement during clinical clerkships such as OB-GYN or family medicine. However, IPV content is often brief, isolated, or optional, and rarely vertically integrated or reinforced longitudinally within the curriculum [11].

The literature suggests potential benefits of longitudinal, integrated IPV education, yet most IPV curricula described in the literature remain short-term educational experiences, with only a limited number of programs incorporating IPV education consistently across the broader medical school curriculum. Studies report the use of trainee feedback, pre- and post- surveys, and objective measures such as OSCE performance. However, few have investigated the long-term effects of training on clinical behaviors, such as screening. Limited time is frequently cited as a major barrier, along with funding, faculty support, and institutional endorsement as additional obstacles to long-term integration [11].

Other types of IPV training in undergraduate medical education have shown promise but are not the norm within the medical curriculum. One study demonstrated that among 1st- to 3rd- year medical students who participated as educators in a community outreach program on IPV in addition to didactics, students had significantly higher confidence in their ability to address IPV issues compared to those who received only didactic curriculum. Significant improvements included confidence in recognizing forms of abuse, discussing the magnitude of the problem, discussing partner abuse, providing resources for victims, and helping motivate victims to act through exploring their own beliefs [12]. 

Simulation-Based and Standardized Patient Training

A study investigating a simulation-based trauma-informed care curriculum for medical students across four medical schools demonstrated significant short-term improvements in self-reported learner confidence and perceived preparedness. In the study, 17 medical students participated in a didactic session followed by three acute-care simulations, with one simulation case addressing IPV. Post-intervention surveys showed statistically significant increases in self-reported confidence when trauma screening is indicated and inquiring about trauma in a sensitive manner (p<.01) [10].

Similarly, standardized patient-based IPV curricula improve short-term comfort addressing IPV-related discussions, with significant gains in recognizing IPV, initiating screening, and discussing resources. A group of 16 preclinical medical students participated in a didactic session followed by a standardized patient encounter and debrief. Post-intervention surveys showed significant increases in self-reported confidence in recognizing signs of IPV (p<.01), asking about IPV (p<.01), discussing resources (p<.01), and comfort addressing IPV-related issues (p<.01) [13].

Preparedness and Screening Behavior

Despite these improvements, there are persistent gaps in medical student preparedness. In one survey of third- and fourth-year medical students and primary care residents, all participants believed physicians should discuss IPV with patients, yet only 6.4% reported usually or always doing so in clinical practice. Additionally, approximately half reported never or rarely discussing IPV with patients at all [14].

Assessment of Long-Term Outcomes

Evidence regarding long-term retention and sustained behavioral change remains limited, with many studies relying primarily on short-term self-reported outcomes rather than objective assessments of clinical competency [15].

Discussion

Despite participation in existing IPV educational interventions, many medical students enter clinical rotations without sustained confidence in applying IPV knowledge to patient care. Simulation-based and skills-based IPV interventions have demonstrated short-term improvements in student knowledge, confidence, and communication skills immediately following training [15,16]. However, these gains are often measured using immediate post-intervention assessments, limiting conclusions about long-term retention of skills or preparedness for subsequent clinical encounters [16]. Additionally, existing interventions vary widely in content, duration, assessment methods, and outcome measures, making it difficult to compare effectiveness across programs or establish best practices [11,15]. This inconsistency contributes to gaps in perceived readiness among students, particularly when IPV education is not clearly linked to anticipated clinical roles or reinforced prior to clinical clerkships [7,8].

Greater exposure to IPV training has been associated with increased perceived knowledge and preparedness, which in turn correspond to greater perceived readiness when addressing IPV-related patient interactions [9,16]. Notably, models that extend beyond traditional didactics, including community-based IPV outreach programs in addition to receiving standard coursework, show promise in addressing these gaps [12]. Together, these limitations underscore the need for a structured, longitudinal, and community-based preclinical IPV education intervention that is intentionally timed, clinically relevant, and designed to improve medical students’ readiness prior to entry into clinical rotations.

These educational gaps are not only a failure to meet intended curricular learning outcomes, but may also contribute to missed opportunities for patient identification and intervention; studies of real-world practice demonstrate persistent under-screening for IPV despite frequent patient contact. As previously stated, out of 154 patients presenting with chief concerns warranting IPV screening, only nine were appropriately screened, highlighting a substantial gap between clinical presentation and provider action [5]. These findings suggest that inadequate provider preparation may contribute to missed opportunities for patient care despite frequent screening opportunities within the patient population, suggesting current approaches may overestimate preparedness by emphasizing confidence rather than consistent clinical behavior. Future research should evaluate longitudinal and community-based IPV education using objective measures of screening behavior in clinically realistic environments rather than relying solely on self-reported confidence.

## Conclusions

In conclusion, early exposure to IPV education during preclinical training plays an important role in normalizing screening and establishing foundational comfort. Introducing IPV concepts early frames screening as a routine component of patient care rather than a sensitive or exceptional task. Skills-based, trauma-informed training may help address the gap between theoretical knowledge and clinical readiness. Because IPV is highly prevalent in reproductive health contexts, future work should explore integration throughout the reproductive pre-clerkship curriculum and within clinical contexts where students are most likely to encounter and screen for IPV. Overall, while IPV education appears to improve short-term confidence, existing evidence suggests that longitudinal, community-based, integrated approaches may be more effective for sustained impact, while current approaches may overestimate preparedness by emphasizing confidence without consistent application of screening behaviors in clinical practice.
